# A User-Centric Knowledge Creation Model in a Web of Object-Enabled Internet of Things Environment

**DOI:** 10.3390/s150924054

**Published:** 2015-09-18

**Authors:** Muhammad Golam Kibria, Sheik Mohammad Mostakim Fattah, Kwanghyeon Jeong, Ilyoung Chong, Youn-Kwae Jeong

**Affiliations:** 1 Department of CICE, Hankuk University of Foreign Studies, Youngin-si 449-791, Korea; E-Mails: kibria@hufs.ac.kr (M.G.K.); fattah@hufs.ac.kr (S.M.M.F.); email@hufs.ac.kr (K.J.); 2 Electronics and Telecommunications Research Institute (ETRI), Daejeon 305-700, Korea; E-Mail: ykjeong@etri.re.kr

**Keywords:** Internet of Things, Web of Objects, knowledge creation model, semantic ontology, user-centric Internet of things service

## Abstract

User-centric service features in a Web of Object-enabled Internet of Things environment can be provided by using a semantic ontology that classifies and integrates objects on the World Wide Web as well as shares and merges context-aware information and accumulated knowledge. The semantic ontology is applied on a Web of Object platform to virtualize the real world physical devices and information to form virtual objects that represent the features and capabilities of devices in the virtual world. Detailed information and functionalities of multiple virtual objects are combined with service rules to form composite virtual objects that offer context-aware knowledge-based services, where context awareness plays an important role in enabling automatic modification of the system to reconfigure the services based on the context. Converting the raw data into meaningful information and connecting the information to form the knowledge and storing and reusing the objects in the knowledge base can both be expressed by semantic ontology. In this paper, a knowledge creation model that synchronizes a service logistic model and a virtual world knowledge model on a Web of Object platform has been proposed. To realize the context-aware knowledge-based service creation and execution, a conceptual semantic ontology model has been developed and a prototype has been implemented for a use case scenario of emergency service.

## 1. Introduction

The user-centric service features in the Internet of Things (IoT) [1] represent an important part of daily life that maximize and optimize multiple types of user satisfaction. A semantic ontology-based service environment in the IoT infrastructure helps in the decision making to maximize the user-centric services through the knowledge stored in the IoT user data repository.

The service creation of cross-domain applications to simplify the objects as well as application deployment, maintenance and operations are facilitated through the application level common platform—Web of Objects (WoO) [2]. Technical goals, such as open homogeneous distributed service infrastructure achieved by the WoO will contribute to the improvement of service features that deal with big data. Modifying the data to be used for information reusability and connecting the relation of information to provide intelligence should be considered. Thus, a semantic ontology is applied to the provision of knowledge-based services.

The WoO supports semantic modeling of objects that provides a user-centric IoT service model with the use of a semantic ontology. The semantic ontology maintains the relationship among all the virtual objects (VOs) for information reusability. WoO refers to the objects that are connected to the internet, and broadly applied through the web that expose the function as a method to approach a particular object. WoO combines the characteristics of web applications along with the VOs, where application development and flexibility of access from the web are integrated. VOs have been designed to support dynamic composition and orchestration of functions to create the user-centric service environments.

To satisfy user-centric service requirements, context awareness plays an important role. It is necessary to convert the real world data into meaningful information to make decisions based on the context. Attributes and properties of the physical devices and information are defined using a semantic ontology to form virtual objects on the WoO platform, so that raw data collected through these devices (such as sensors) can be used as well as stored in a database to be reused later. Connecting the relation of the information to form knowledge provides intelligence to the IoT environment. The semantic ontology enables the relation of the information by sharing and merging among them to form knowledge, where knowledge is captured, processed, reused and communicated semantically in a knowledge-based system. Storing and reusing the objects in a knowledge base can be expressed by semantic ontology that defines common vocabulary to share information in a domain.

To provide intelligent service features in an IoT environment, both the context and knowledge of the previous services should be considered thoroughly. In [3], authors have presented a decision support tool to provide intelligent service features based on the context, where previous incident logs have been considered as the basis of the tool. Since objects have been defined using a semantic ontology, all the incident logs have been collected through the VOs and stored in the log repository for future use, but due to the absence of composite virtual objects (CVOs) the system lacks a context-aware knowledge-based service composition mechanism. To overcome this lack, this paper focuses on a virtual world knowledge creation model that synchronizes the virtual world knowledge model and the service logistic model to develop a knowledge-based smart IoT environment. Also, the creation of CVOs and a context-matching algorithm are presented in this regard. To realize the user-centric knowledge-driven IoT services, a use case scenario of an emergency service at a smart shopping mall has been implemented and demonstrated.

The rest of this paper is structured as follows: Section 2 presents related works. Section 3 discusses the representation of real world objects as virtual world objects, and the CVO creation and activation process. This section also introduces the functional layered architecture of the WoO platform. Section 4 presents semantic ontology provisioning in terms of service model, device model and resource model. Section 5 describes the context awareness and service composition process on the WoO platform. Section 6 focuses on the knowledge base, its components and a knowledge creation model to provide user-centric service intelligence. Section 7 presents the prototype implementation in the use case of an emergency service. Finally, this paper concludes in Section 8.

## 2. Related Works

Related research works have been undertaken to incorporate applications and services into the virtual world. The real world physical objects, such as physical devices, need to be virtualized to be accessed in the virtual world. Virtual objects (VOs) are harmonized and composed to form application level features to satisfy the user-centric service requirements. Object virtualization plays an important role in the WoO platform. WoO-based objectification, virtualization and composition of VOs for information reusability, extensibility, and interoperability among the VOs have been discussed in [4,5,6]. Object virtualization makes it possible to emulate real world objects, so that any operation can be performed on objects in real cases, where both the physical and non-physical objects are virtualized. VOs interact and are interconnected using the semantic ontology presented in [7,8,9].

Combining and coordinating a set of services with the purpose of achieving functionality cannot be realized through existing services. Service composition can be done based on the related VOs, policies and user preferences, where composition architecture, methodology and algorithm have been presented in [10,11,12]. Composition concerns synthesizing a specification of how to coordinate the component services to fulfill the user request. A desired behavior can be achieved by combining the abilities of multiple services by service composition. On the other hand, orchestration is about executing the result of composition synthesis by coordinating the control and data flow among the participating services and also about supervising and monitoring that execution.

In the WoO, objects are applied though the web for easy composition of services. Web service composition is categorized as either manual or automated. Automated web service composition allows limited defined user requirements for a desired composite service. In [13,14] the authors proposed architectures for semantics-based, context-awareness, and dynamic service composition. They introduced CoSMoS, a semantic abstract model for both service components and users, which is the basis for all required representations of their framework. Their composition approach involves receiving a natural language request from the user, which is parsed using preexisting natural language analysis technologies into a CoSMoS model instance.

Networked intelligent objects offering smart behavior as web services will lead to a service-oriented infrastructures. In a smart IoT environment, smart devices can sense their internal state and are able to communicate through data networks, but sensor networks can measure both the internal and external state of the environment. To provide the intelligent IoT environment, Service Oriented Cross-layer infRAstructure for Distributed smart Embedded devices (SOCRADES) presented an architecture to integrate these smart objects to fully accommodate user demand in [15]. SOCRADES SIA (SOCRADES Integration Architecture) from the concepts of SOCRADES provides functions for monitoring and managing the embedded devices in an automated industrial system and has been presented in [16].

Automatic modification of the system behavior according to the current situation with minimal human intervention is possible with the context awareness. Due to the rapid changes of user context, use of context information is important in context-aware applications. The context and its nature in context-aware computing have been surveyed in [17]. Once the context is known, service rules can be applied to create intelligence. A user context offering context-aware services and applications to build better service provision to the user have been proposed by BUTLER project in [18]. IoT-related use cases to provide context-based services by using semantic ontology model have been discussed in [19,20]. In case of changes in the context, the system compares and matches to make decisions, where a context-based decision support system has been considered. References [21,22] have presented the system designed by the SENSEI project to provide network and information management service for enabling reliable and efficient context information to be used in wireless sensor and actuator networks.

To provide knowledge-based IoT service, knowledge needs to be captured, stored and reused for the system to be more experienced. Knowledge is perceived as meaningful information or the understanding, awareness, and familiarity acquired through study, investigation, observation or experience over the course of time. Knowledge management is concerned with exploitation and development of knowledge of an organization. Collected knowledge can be organized by indexing the knowledge components, filtering based on contents and relationship among the objects. References [23,24,25,26,27] have described knowledge creation functionality, methodology and components associated with knowledge-based systems. Objects that need to be captured and stored in a knowledge-based system are expressed using a semantic ontology.

Identifying and combining the most relevant objects to be combined to offer appropriate services in a smart environment is a complex task in a user-centric service environment. The cognitive management framework in IoT dynamically changes the real world objects into a virtualized environment, where cognition is used to select the most relevant objects for the purpose of an application in an intelligent and autonomic way. Cognitive entities at all levels provide the means for self-management, such as self-configuration, self-optimization and self-healing and learning. The abstraction of technological heterogeneity derived from a vast amount of heterogeneous objects and three fundamental processes of this framework, such as dynamic CVO creation, knowledge-based CVO instantiation, and CVO self-healing have been addressed by the iCore project in [28,29]. A cognitive management framework and associated functionalities for IoT have also been proposed.

## 3. Smart Virtual World Creation in the Internet of Things (IoT) with Web of Objects

Even though IoT visualizes a world where various intelligent objects share data with each other or cooperate in groups to achieve complex goals, it faces a lack of common standards both at the network and application level. To overcome this lack, WoO facilitates smart distributed applications that combine isolated information from different domains. In order to facilitate simple development, deployment and operation of smart distributed applications, an integrated design based on a uniform resource efficient infrastructure, uniform data and service models is required. WoO facilitates the easy creation of cross-domain applications able to target goals that have not been envisaged at system deployment time.

It is always not possible that physical devices are directly available to the main applications. Providing real time information about any process running remotely that consists of physical devices like sensors is difficult. Because of the limited resources of sensors, they cannot sustain long communication sessions. Also, if there is large number of requesters, a resource-constrained object may not be able to handle such a large number of requests simultaneously. In such a situation, a convenient solution is to provide those resource-constrained devices and their functionalities as VOs through some capable devices. WoO allows physical devices and information to be represented as VOs that are harmonized and composed to form application level service features.

**Figure 1:**
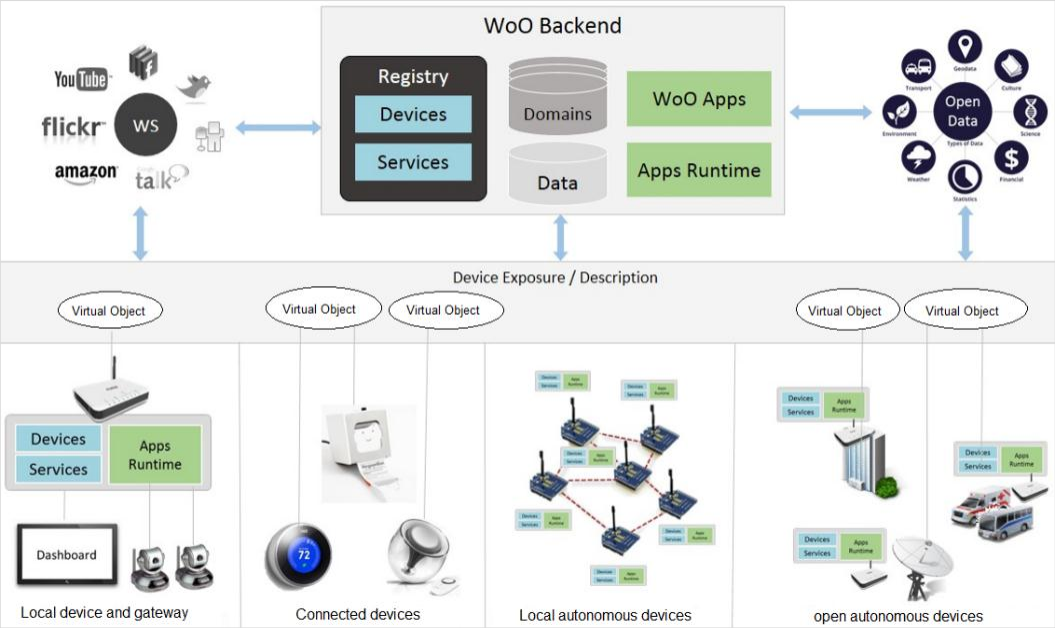
Real world physical objects, such as physical devices, gateways, local and open autonomous devices are connected and virtualized in the WoO concept [2,3].

The concept of WoO shown in Figure 1 includes two functional levels: local and backend level. Objects connection, capabilities discovery and features exposer are the concerning factors in the local level. Design, execution and support of IoT-based applications are supported by the WoO backend that interacts semantically with open data.

With virtualization, it is possible to emulate the real world device as it is, so that any operation can be performed as in a real case. Rather than providing access to the device via a modified interface, the VO concept can provide a better view of the device. Devices such as sensors or other physical devices actually represent real world objects, and virtualizing the device can exploit and provide access to the virtual world. VO is associated with service parameters, such as a temperature sensor in a room that can measure the room temperature.

VOs are virtual representations of features and capabilities of devices and information in the virtual world, but they hide the underlying technological heterogeneity. Communication among the devices requires object virtualization. Object abstraction and heterogeneity derived from diverse physical devices and information ensure the object virtualization. Virtualizing objects via a gateway has become a common solution in the field of sensor and actuator networks. With this approach, the data processing load can be shifted from resource constrained objects to resourceful ones. Better quality of service can be maintained in service provisioning and data access.

VOs are stored in a VO repository that contains information including the VO identifier, location, associated functions and access rights. VO information is defined in a Resource Description Framework (RDF) into an XML file to be represented semantically. RDF represents the metadata of web resources and is stored in an RDF graph database that implement the VO database. VO communicates with the physical devices through the gateway using a REST interface.

**Figure 2:**
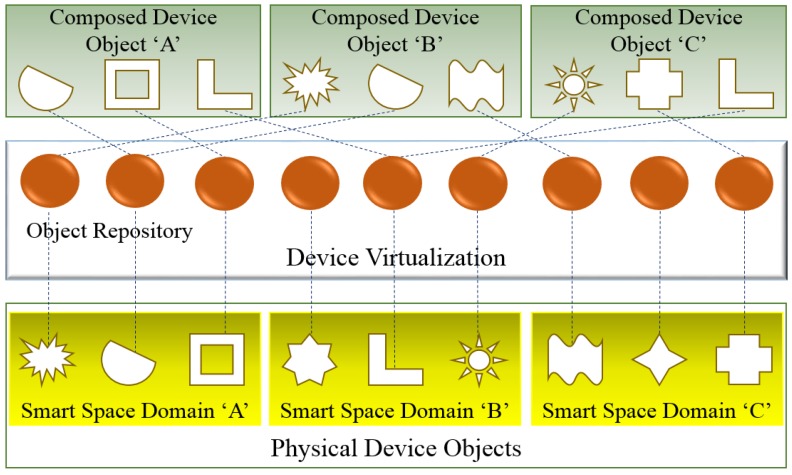
Composition, collaboration and harmonization of VOs for services composition.

Combining the VO information and attributes for service composition in WoO increases the scope of IoT services. The user-centric service models can be defined from the service description through the collaboration and harmonization among multiple types of related objects stored in the object repository. WoO allows objects to collaborate and be harmonized in a semantic ontology. Figure 2 shows the composition, collaboration and harmonization of virtual objects.

**Figure 3:**
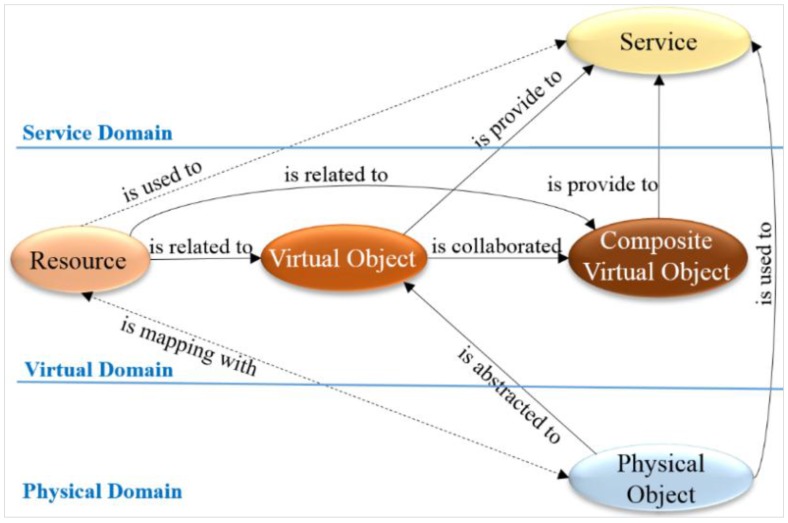
The object relationship model in the WoO platform relates physical objects and resources to provide services [2,7].

Interaction between internal and external VOs is the key aspect in the WoO. Interaction between internal and external VOs is possible through the interconnected physical devices and semantic information. Object relationship models define the main abstractions and concepts that describe the relationships between physical object, resources and services. The main principle of the WoO is extension of the web with the physical world into the virtual world, to involve interaction with physical entities in the ambient environment. Three layers on the WoO platform, including the physical domain, virtual domain and service domain, are shown in Figure 3.

Resources are the necessary elements, implemented to simply abstract the physical object to control devices in the real world. Resources are used to create services along with the provided VOs. Resources are related to VOs that are combined to compose the VO. Multiple VOs are combined to form a CVO that represents user-centric services in the service domain. With the separation of domains from the physical object, VO benefits the flexibility of VO creation considering information in the IoT service environment. Section 4 describes the services, devices and resource models using a semantic ontology.

### 3.1. Model for Composite Virtual Objects (CVO)

The CVO is one of the three levels of functionality that meet the goal of service provisioning on the WoO platform. Multiple interoperable VOs and service rules are combined to create a CVO. At this level, information of VOs and available functions are retrieved from the VO repository. The user-requested service is realized in terms of created CVO that shares and merges information and functionalities of defined VOs. The created CVO is stored in a CVO repository at the CVO level so that the service might be offered directly if the context of the service matches with the stored one. The CVO repository contains information of the stored CVOs, including the identifier of the CVO, access rights of the CVO and VOs identifiers. The CVO is able to provide an optimal way to provide application-level services if the capabilities of VOs and application requirements are available. For example, ProductAdvertisement is a CVO that can provide sale information of specific products to the customer in a use case scenario at a shopping mall while the customer is passing by a shop or zone; here Customer, Product, priceOfProduct, saleInfo and/or season are the interrelated VOs.

Heterogeneously distributed networks are the challenges of offering a knowledge-based service. In the WoO, all of the devices are objectified and virtualized, where contextual facts can be inferred from the defined VOs, CVOs and rules. Reference [29] has proposed a cognitive mechanism for CVO creation and knowledge-based CVO installation, where cognition is used to decide the interrelated VOs and CVOs that meet the application requirements. Cognition is the process of problem solving and decision making by evaluating and reasoning the surrounding knowledge that focuses on the representation and transformation of information. Components of cognitive management including VOs, CVO and the knowledge base have the ability of self-management and self-configuration to offer intelligent services. The concept of cognition on the WoO platform is applied by the creation and installation of CVOs through the virtual representation of interoperable and interconnected VOs semantically, where cognition is achieved by inferencing those VOs and CVOs. Components of the cognition, such as CVO creation, context matching and knowledge creation model will be elaborated later.

**Figure 4:**
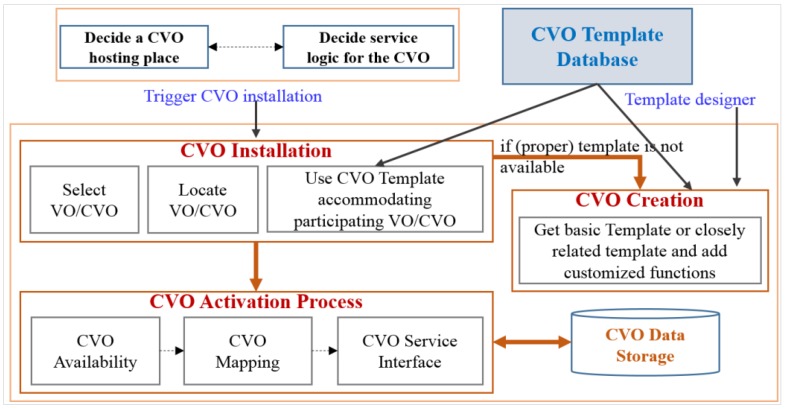
CVO creation and activation process at the CVO level to offer user requested services.

The CVO leads to intelligent services, and fulfills requirements, while the details and complexity are hidden from end users. CVO framework components exploit cognitive mechanisms to enable the mashup and reuse of the existing VOs and CVOs by various applications. Besides the attributes of the created CVO, it inherits all the functions and features of interrelated VOs. A CVO combines the function and features of VOs for the provisioning of services by maintaining orchestration with other related objects. According to the user requirements, multiple interrelated VOs and CVOs are composed to provide application-level services. The developer creates CVO templates based on the service requirements, policies and available VOs that are stored in the CVO database. Creation of a CVO requires some criteria. The CVO instance is created from a defined CVO template according to the user requested service. In the CVO installation, the candidate CVO needs to be selected and located as per the service requirements. The participating VOs and CVOs are collected from the VO and CVO template database, respectively. The CVO installation is followed by the CVO activation process. The CVO creation and activation processes are shown in Figure 4.

In the CVO activation process, the CVO manager searches for the available CVO template in the CVO database and, if found, then it is provided directly. The framework components are responsible for searching the predefined CVOs. The reuse of existing CVOs increases the efficiency of computation and time and leads to resource savings. The CVO search mechanism follows a matching algorithm based on some rules and threshold values that are predefined. If a proper CVO is not matched, then the CVO manager collects associated VOs for CVO creation; in this case the CVO manager sends a request to the VO manager for providing appropriate VOs according to the virtual world information. The associated VOs, service policies and user preferences are inferred with the inference engine.

### 3.2. Functional Architecture of the Web of Objects (WoO) Platform

**Figure 5:**
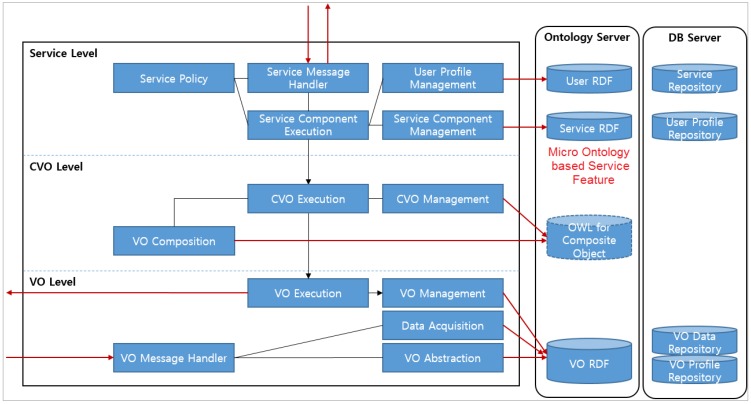
Functional layered architecture of the WoO platform leverages applications in the IoT in accordance with less complex resources and real world objects [2].

Various IoT-related projects that provide services appear to have limits in their provision to meet the user-centered service capability. In order to solve the limits in provision of orchestration in IoT services, a WoO platform is strongly requested to support flexibility in service integration and management, location and context management. The functional architecture of the WoO platform consists of three functional levels: service level, CVO level and VO level, as shown in Figure 5.

In order to support the features, a WoO should be much more resource aware. A WoO provides mechanisms allowing objects to be aware of and to react to their environment. Semantic ontology is applied to describe objects, their capabilities and mechanisms as well as expose and manage the objects on the web with respect to existing regulations. It also adapts existing embedded service technology to the specific requirements of resource-constrained devices.

Moreover, through semantic ontology the relational information is delivered by accessing the main database. The WoO service platform supports a dynamic mechanism to compose the user-centered IoT service in conjunction with the semantic ontology indicating a partial or full relationship among VOs. Thus, the orchestration functions for user-centered IoT services on the WoO platform could be designed through a semantic ontology web service concept. The WoO specifies and develops mechanisms for creating, composing, deploying and managing objects, and provides an aggregation of services usable in applications. The WoO also provides a way to integrate legacy systems in the IoT environment.

In the WoO architecture, the VO level is connected to a smart gateway and performs message queue functions and message interpretation. Physical devices and information are virtualized at the VO level to form VOs that are stored in the VO database. The VO execution module at the VO level provides the interface functions to interact with the CVO execution module at the CVO layer to handle requests from the CVO level. The WoO platform uses the attributes of devices to create VOs. According to the user request, various devices are objectified. In that way, by connecting VOs, the user expects that WoO-based IoT services are created and provided. Through the semantic ontology, VOs are simply defined according to the service ontology and service RDF. Accordingly, various physical devices are easily mapped with personal VOs through the web.

The service level provides the necessary functionalities for interactions between the associated VOs and CVOs, which are processed using a web interface. The web service platform possesses versatility and scalability where multiple users or basic environment could easily be applied. The service level is assigned executable algorithms, policies and logics that are to be provided to the users.

The WoO functional architecture provides automation, interoperable by human and machine interpretable representation. The service model in the WoO domain is suited to adapt ontologies that model devices and services. These may serve as a high-level model that references and builds upon existing vocabularies such as sensors, observation, measurement and location from several micro ontologies as well as integrates and merges contents of web as a global repository. The features provided by the WoO include configuring various things into an object that could ease the building of applications, applying the benefits of web features in IoT service provision (e.g., bookmarking, caching, linking, searching, securing *etc.*) and enabling mashups. Necessary components to provide the features of WoO include:

- Semantics ontology: for intelligent data processing using ontology and semantic-level handling
- Context awareness: detection of environmental change which triggers next actions
- Knowledge base: based on the knowledge, application should make intelligent decisions that pave the way for further actions

## 4. Semantic Ontology Provision

A semantic ontology is the study that classifies each object in the real world that might be or not exist in a domain. The ultimate goal of the semantic ontology is to extend the semantic web as a universal repository of all types of data, information and knowledge through classifying, integrating, sharing, merging and using every object connected to the internet [30].

The information that has been expressed by a semantic ontology within the system can be shared among the objects automatically. For sharing and merging information, it is important to use the same names and definitions for the same type of entities. Thus, following the terminology standardized by some standard bodies would be the solutions. In the use case scenario, we tried to use the same terminology to define objects, such as Person, Location, Device, *etc*. Several benefits of using a semantic ontology on a WoO platform include common format RDF, hierarchies of classes and subclasses to identify primary and secondary context types, data integration across applications and information sharing among objects.

In a WoO, the heterogeneous abstraction of objects with the VO and CVO can be achieved in a uniform way of representation through a semantic ontology. The semantic ontology is applied in the WoO to create a user-centric service model in terms of VO and CVO. The semantic ontology is used in object specification, description, object registration and discovery for defined VOs and CVOs. Thus, the semantic ontology can ensure the flexibility of service provisioning in terms of availability, performance, and dependability. At the beginning, attributes and properties of physical and non-physical devices are described in RDF/XML format, thus devices are virtualized in the semantic ontology to be controlled virtually as real world objects. In the WoO, multiple related VOs are mapped together to form CVOs at the CVO level. The semantic ontology uses a reasoner to check the validity of the domain and infers with associated VOs, CVOs, rules and threshold values according to the application requirements.

The semantic ontology is described using Web Ontology Language (OWL) [31], characterized as a major formalism for the design and dissemination of ontology information, particularly in the semantic web. The OWL is implemented into semantic editors such as Protégé [32] and semantic reasoners such as Pellet or Hermit. OWL ontologies provide classes, properties, individuals, and data values and are stored as semantic web documents. OWL ontologies can be used along with the information written in RDF [33]. RDF can be used to model metadata about resources residing in the web, such as title of the resource, its authors, date of creation, modification, and so on.

### 4.1. Semantic Description of the Service Model

**Figure 6:**
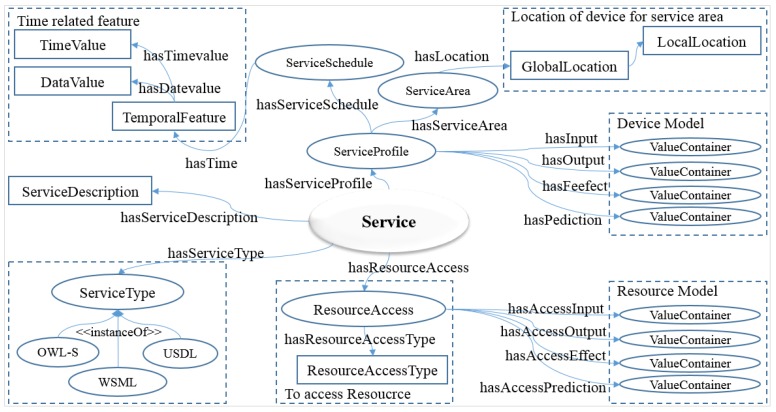
Service model in semantic ontology that describes the profile of service semantically.

To describe the semantic profile of the service model and to share the related information among the devices and resources, objects in a WoO have been described in the semantic ontology model. The service may have several descriptive properties such as input and output preconditions and post conditions, and additionally includes nonfunctional properties such as quality of service. Service provides a well-defined and standardized interface, offering all necessary functionalities for interacting with devices and related processes. The service exposes the functionalities of a device by accessing its hosted resources. The needed access interface of a device is provided during the service provision by including the information of mapped resource model with device model. Figure 6 shows a service model according to the semantic ontology, which is the logical structure on WoO service platform.

The service domain provides a different combination of objects in the virtual domain in order to compose one necessary service in different forms. It may take up objects whether they are VOs or CVOs to create suitable service provision. In order to provide a semantic ontology-based service through a resource model, the appropriate resource information can be accessed. As the resources of service are mapped with devices based on resource information, the appropriate devices are capable of being controlled and managed.

The WoO service model is composed of properties including ServiceDescription, ServiceType, ServiceProfile, ResourceAccess, ServiceSchedule and ServiceArea. ServiceDescription, moreover, includes information to identify the service name, ID and ServiceCategory. ServiceType defines the service explanation of modeling language type technique. ServiceProfile defines the profile of service, range of service, schedule, input and output, precondition, effect and other related information. It also relates to the device’s ValueContainer in the device model. ServiceSchedule defines the date and time. ServiceArea defines the global and local location of device.

The semantic description of services from the virtual domain eases the automatic service management, composition, requests and so forth. A semantic ontology is a formal representation of a set of concepts within a domain and the relationships between those concepts. Some advantages of semantically described services are:

- Applications that use the services can get the profile and features of the services from an ontology repository, thus being able to choose the most appropriate, according to specific constraints
- The service composition is easily enabled and the generation of new services based on already existing ones is a new feature
- Know exactly the context in which services are provided
- Control the function of the service

### 4.2. Semantic Description of the Device Model

**Figure 7:**
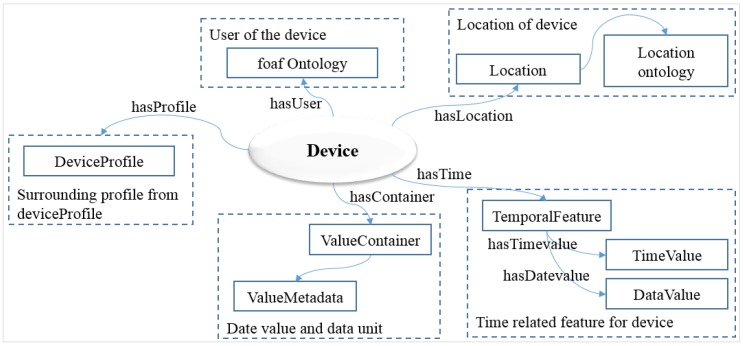
Device model abstracts a physical device using the semantic ontology to provide device profile information to the service domain.

The service, device and resource domains are interconnected to each other on the WoO service platform. The device domain provides device information and the resource domain provides resource information to the service domain, where information of device and resource are mapped to each other. The device model defined using a semantic ontology includes properties that describe the device shown in Figure 7.

Location information is defined in the location ontology that has the same identical model used in the resource model. TemporalFeature indicates the time and the date specified as TimeValue and DateValue. User information is applied in the form of an ontology. The model also includes a DeviceProfile that defines the profile of the device and ValueContainer that contains one or more values of the device. ValueMetadata specifies the information of units that is used for the data value stored in ValueContainer.

### 4.3. Semantic Description of the Resource Model

**Figure 8:**
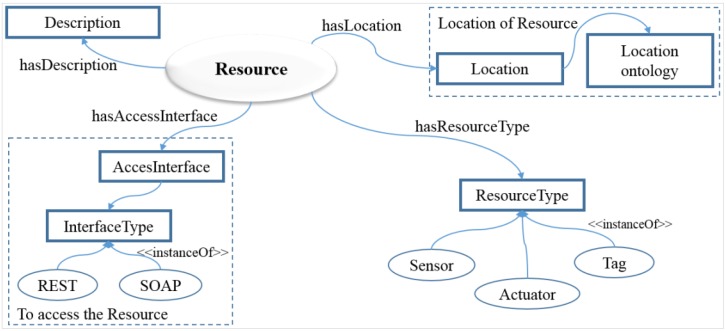
The resource model defines resource information in the semantic ontology to provide resource information to the service domain.

The resource domain defines the access information of resources, such as interface that is used to access to physical devices and protocol. Resource and device models are connected to each other through the service domain. Figure 8 shows the resource model defined using a semantic ontology that controls the device in accordance with the classified model.

The resource model could provide the core components of the related resource data to the device model and service model to make effective and efficient decisions. The resource model includes AccessInterface that gives access to the interface that specifies what technology interface to use in the InterfaceType. Location defines the location of the resources which is mapped by the location ontology and has the same model used in the device model. Description properties define the description of the resource and ResourceType specifies the types of the resources, such as sensor, actuator and tag that could provide information. InterfaceType further specifies the set of instances that reflect the interface technology, such as REST and SOAP. Device and resource models are implemented using a semantic ontology to abstract the physical devices as well as to control them according to the resource model. These models are interrelated with each other to create VOs on the WoO platform that enable flexible creation of user-centric IoT service model.

## 5. Context Awareness and Composition in WoO

In context awareness, context means the meaningful information that characterizes and identifies the surrounding situation of objects, such as persons, location, time, physical or non-physical devices that are coordinated and interact among each other according to the application and user requirements. Context awareness originated from the ubiquitous computing notion that deals with linking changes in the IoT environment. Applications can adapt the context by exchanging information among objects to make more informed and intelligent decision.

In WoO, objects have been virtualized using a semantic ontology to form VOs that have the ability to detect implicit information to automate the applications. In use case scenario, different objects including primary context types, such as Location, Floor, Gateway, Bluetooth and Sensor have been defined to collect actual context information to characterize the situation of an entity. The primary context types are used to identify the secondary context type, such as Location’s information, Floor information *etc.* that allow hierarchies of classes and subclasses in the semantic ontology. It is also necessary to categorize the features of context-aware applications. In use case scenario, information based on available context has been retrieved by the associated VOs (such as TempSensor, HumSensor, CO Sensor and CO_2_ Sensor) automatically that has been classified as automatic contextual reconfiguration.

In a user-centric IoT environment, context awareness plays an important role in the pervasive computing architecture to enable the automatic modification of the system behavior according to the current situation, time and location with minimal human intervention. Context has a powerful and long-standing concept in human machine interaction. As human beings, we can interact with each other more efficiently by fully understanding the context in which the interactions take place.

**Figure 9:**
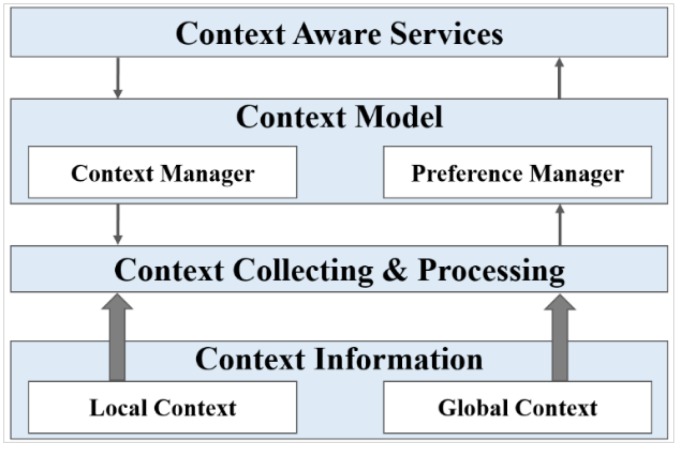
General context-aware architecture enables the system to optimize services by utilizing the available context information.

In a context-aware IoT environment, two types of contexts, such as local context and global context, are considered to provide intelligent services. The state of the objects, such as state of the resource is represented by a local context that is inferred locally. Exchange of local contexts between multiple objects is required by the global context that includes the system level context, such as user context. Collecting and processing of these contexts are handled by the context manager at the context level to make appropriate decisions. The preference manager provides user preferences compared with the previous history to provide context-aware services. Figure 9 shows the general context-aware architecture. In the scope of a user-centric IoT environment, users, devices and environment contexts are considered in order to bring more efficient service composition.

The semantic ontology makes context awareness possible by relating and sharing information among the objects. In a context-aware IoT environment, high level context information is generated from the low-level raw data collected from different related objects, such as users, sensors, location and time of the system. In our use case scenario, the status of the shopping mall has been classified by the application server based on the context information provided by objects via a gateway, provided context information has been inferred to make decisions, thus satisfying context awareness. In the WoO, the reasoner used in the semantic ontology checks the validity of the defined model and applies defined rules and axioms to offer services, thus services are provided even though physical devices are not connected directly to the user application.

### 5.1. Context-Aware Service Composition

**Figure 10:**
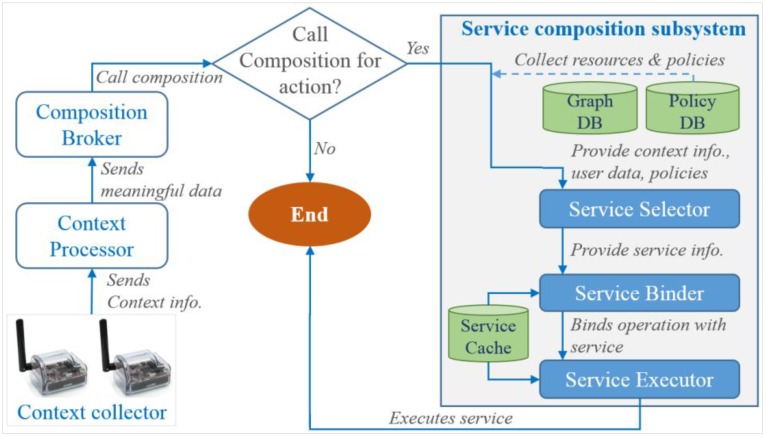
Composition process based on context information provides efficient and accurate service composition.

In the context-aware service composition process, context information is collected by sensors that are classified as context collectors. In this process, a composition broker acts as the composition decision maker, where a composition subsystem is in charge of answering composition requests from the composition brokers with regard to collected context information. The ontology and graph database provide the semantic database. The flow diagram of the context-aware composition process is shown in Figure 10.

The process starts with signals from the context collectors by detecting changes in the context. Context collectors send context data to the context processor. The context processor processes this data into meaningful and machine readable data, which is then sent to the composition broker to decide whether to move on by calling the composition or not. In case no action needs to be carried out, the system switches to the sleep mode or ends, otherwise the composition is called. In the next step, resources are collected from the database to support the composition process. The service selector uses the provided context information, user data and policies to infer appropriate services as well as creates a concrete description of the required composite service. The service binder follows up by binding the operations of the selected service, where the service executor gets access to the service cache to execute those operations.

To provide context-aware services by the applications, it is necessary to match the right context at the right time due to the continuous change of the user context, additionally services might vary for different context at different time. Based on the context information it is easier for the human to match the context to decide an appropriate service manually, but harder for the system. Thus all the possible real world objects, locations, services, activities *etc.* are defined as classes and subclasses using a semantic ontology so that objects can be mapped to each other. Since objects are virtualized and mapped in the WoO, service could be provided by matching the context of the service in terms of VO, CVO, location and time with the history data. Due to collecting, storing and reusing the real world information semantically, context matching in WoO is an important part of providing user-preferred service that will enhance the knowledge. Even though accurate matching of context is not possible all the time, current context information, VOs, CVOs, service rules and threshold values are inferred to offer services.

**Figure 11:**
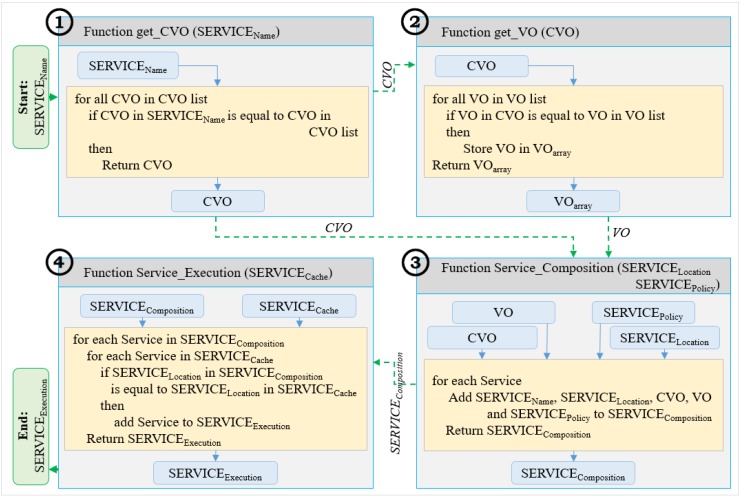
Context-matching algorithm based on location context information.

For the context-aware service composition, a context-matching algorithm has been used. Due to the virtualization of the location object using a semantic ontology, the system supports for the multiple locations. For the simplicity, only the location context has been considered to match the service in the algorithm. Figure 11 shows the sequence of the context-matching algorithm that comprises four functions including get_CVO, get_VO, Service_Composition and Service_Execution. The dotted arrow shows the parameter passing from one function to another in the figure. Service name, location, policy of the service and service cache are the input parameters of these functions. The algorithm starts with the get_CVO function that provides the CVO name associated with the requested service (SERVICE_Name_) and ends with the Service_Execution function that provides the matching service (SERVICE_execution_) from the service cache according to the location context information. At the beginning, SERVICE_Name_ is passed as an argument to the get_CVO function that searches related CVO in the CVO list. The get_VO function searches multiple VOs associated with the CVO collected from the get_CVO function and stores in an array. Then the Service_Composition function composes the requested SERVICE_Name_, SERVICE_Location_, the associated CVO from get_CVO function, VOs from get_VO function and SERVICE_Policy_ by adding to an array and returns the composed service (SERVICE_composition_). Finally, the Service_Execution function matches the service based on the location context and returns the matching service (SERVICE_execution_). Service_Execution function considers the location of the service and service cache as arguments. If the location (SERVICE_Location_) of the service in service composition (SERVICE_composition_) matches one of the location of services (SERVICE_cache_) in the Service Cache, then the service (SERVICE_cache_) is added to an array called SERVICE_execution_, which is then returned by the Service_Execution function for the service execution.

### 5.2. Service Composition on the WoO Platform

**Figure 12:**
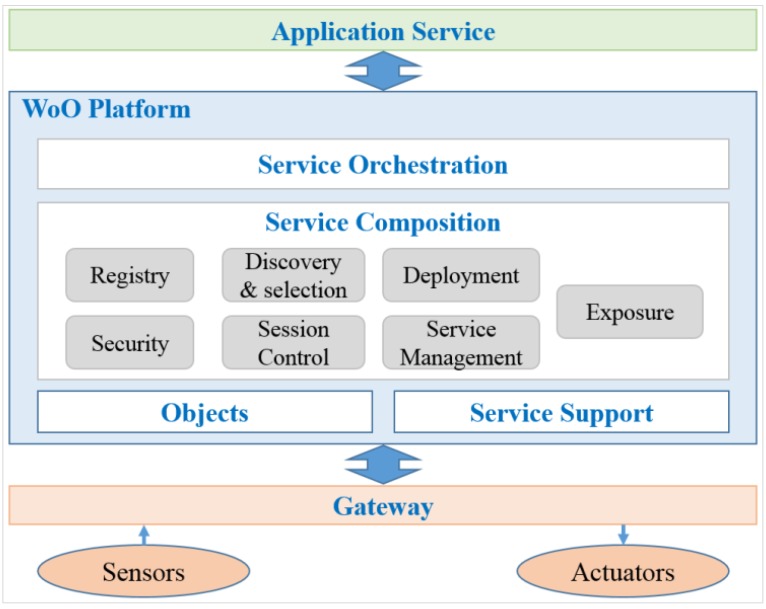
Functional architecture of WoO platform for service composition.

In the WoO, service composition brings underlying services all together to create a new generation of smart applications over intelligent networked devices. By facilitating the framework for dynamic service composition in the domain of WoO, relevant data such as context information can be easily collected, processed and used by the applications. Additionally, with the help of semantic modeling, requirements and descriptions of the applications can be integrated with more user friendliness for communicating among the applications. The functional architecture of a WoO platform for service composition is shown in Figure 12. The architecture consists of service orchestration, service composition, objects and service support. The service composition includes some basic functions such as, registry, discovery and selection, development, security, session control, service management and exposure.

The registry function describes and publishes the offered functionalities of the service to the potential user, a service registry allows organizing information about service and provides facilities to publish and discover services. Service discovery and selection function is the process of locating and gaining access to a provided service that satisfies a set of requirements. The service deployment function involves concretely associating service to devices in the real world system. Depending on whether the service provider or owner decides to expose such information or not is provided by the exposer function.

The security function enables providers to guarantee the delivered service that will respect a given security policy, in any interaction with the operational environment, and regardless of who actually called the service. Session control function deals with the network call control interactions. The service management function comprises all the mechanisms that are required for capturing and processing several attributes of a service execution to determine whether predefined agreements are abided by and the execution goes as planned.

Service orchestration defines which message is sent when and by which participating service. Service orchestration is about executing the result of composition synthesis by coordinating the control and data flow among the participating services and also about supervision and monitoring the execution.

**Figure 13:**
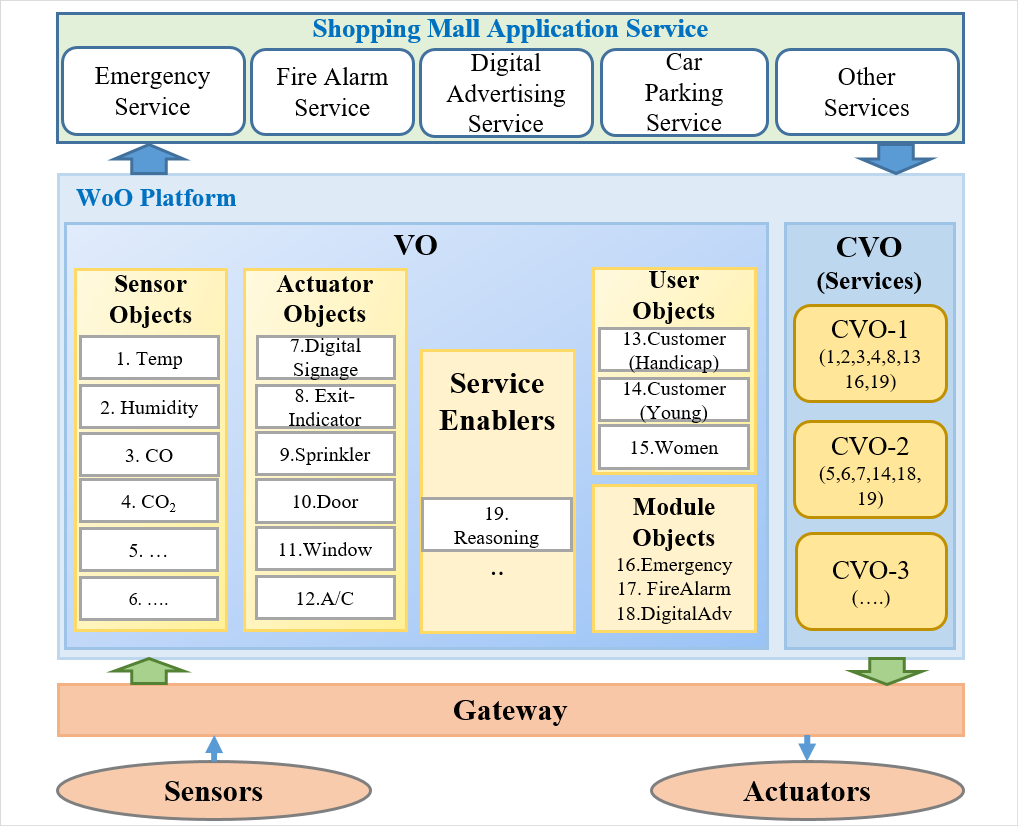
Use case of service composition on a WoO platform.

The architecture of a WoO platform for supporting service composition in our use case scenario of an emergency service is shown in Figure 13. The WoO platform considers all things such as sensor, actuator, service enabler, user, and modules as VOs. For example, Temp, Hum, CO, CO_2_ and CCTV have been considered as sensor VOs; DigitalSignage, ExitIndicator, Sprinkler, Door, Window and so on. have been considered as actuator VOs. Several application-level services include emergency service, fire alarming service, digital advertising service and car parking service. In the figure, numbers are assigned to each VO for easy realization of the composition. CVO converges the VOs and makes a group to create application services. For example, CVO-1 for an emergency service application is created by “composing” Temp, Hum, CO, CO_2_, ExitIndicator, Emergency and Handicap customer.

Service composition to the web involves combining and coordinating a set of services. The composition of services can be performed either manually or automatically based on the context information according to a static composition schema at run-time that can lead to dynamic composition schemas afterwards.

Service descriptions should be written in a formal, well-defined language, allowing for automated processing and verification of the produced description documents. These characteristics are also extremely useful when attempting to compose the web services. Web service description language [34] is an XML [35] format for describing the services as a set of endpoints operating on messages that contain either document-oriented or procedure-oriented information.

## 6. User-Centric Knowledge Creation Model

Knowledge acquisition, representation and applications are the key factors in a knowledge-based system that lies in the virtual domain. The knowledge base is updated by capturing and reusing gathered knowledge. A semantic ontology is a way to represent knowledge in a knowledge-based system in terms of VOs and CVOs on a WoO platform. The knowledge base represents VOs and an inference engine represents logical conditions regarding VOs. A knowledge-based system includes two key subsystems: knowledge base and inference engine.

The knowledge base represents information in a structured way such as hierarchies of classes and subclasses and additionally makes relations among classes. The knowledge base learns and experiences from the individual, social environment, indoor and outdoor activities based on context and schedule. The knowledge is created in terms of four basic levels—individual level, group level, organizational level and inter-organizational environment level. Knowledge regarding individuals or groups is modeled by individuals or groups as a team experience. The team experience is created by the relation with other individuals or groups that is related to the context and conditions by means of socialization. In a knowledge-based system, rules and procedures are applied to the stored data for reasoning new knowledge. The new deduced knowledge is updated and stored in the database, thus enhancing experiences.

### 6.1. Functional Model for Knowledge Creation

**Figure 14:**
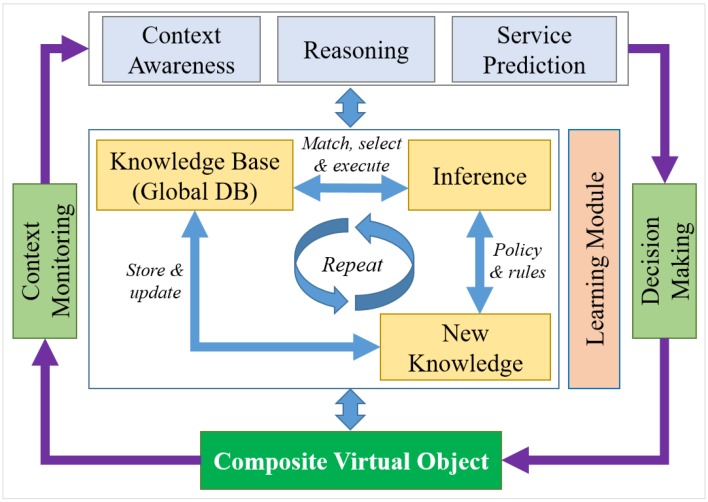
Knowledge-based service creation functionality.

Key components in a knowledge-based functional model include context monitoring, decision making, CVO, inference, knowledge base and learning modules, as shown in Figure 14. The context monitoring is responsible for monitoring the context associated with the request. Context is monitored in terms of context parameters that enable the system to be aware. The context parameters include the information of the schedule, location, user interest, associated VOs and CVOs at a specific time and location. Based on the fact that context awareness means changes in the situation, the CVO performs autonomously. For context awareness, the CVO monitors related parameters.

Upon receiving the request and context parameters, the knowledge-based system matches the parameters in the CVO database to select the available and relevant CVO to be reused. It is not possible to match the same CVO according to the context. The knowledge-based system uses a learning mechanism to identify the most similar one by calculating the Euclidean distance among the CVOs and selects the CVO that has the closest distance at a satisfactory level. If the satisfactory level matching the CVO is not found, the system requests the decision making with the request and context parameter along with the associated VOs. A decision-making tool is used to find optimal CVO composition. It considers the available VOs, functions, policies, threshold values and user preference. The decision-making module interacts with the VO database and collects the VO information, which is sent to the inference engine to run the reasoner to create the CVO. The created CVO is stored in the CVO database. The learning module accumulates the WoO system knowledge, learns the identified context and expected situations. The reasoner translates the application requirements, VOs and policies into a language the system can understand.

### 6.2. Virtual World Knowledge Creation Model in a WoO-Enabled Environment

**Figure 15:**
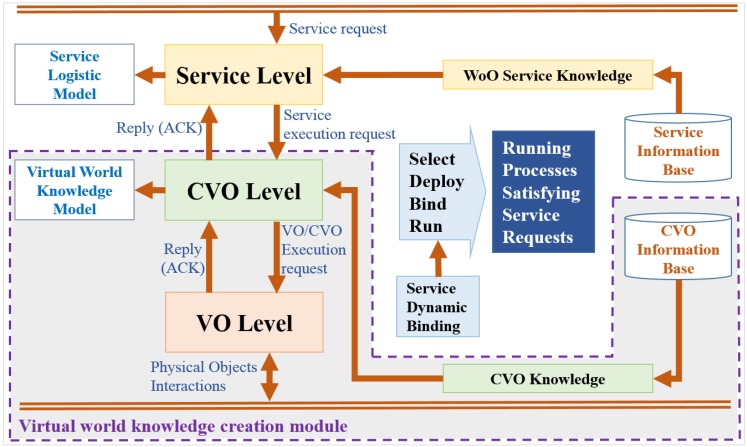
Virtual world knowledge creation model on WoO platform.

Virtual world information is complex, dynamic and always changing, which has a vital role in functioning and executing the service request. The virtual world knowledge creation model in a WoO-enabled environment consists of a virtual world knowledge model at the CVO level that processes the virtual world information and a service logistic model at the service level. The virtual world knowledge and service logistic model are synchronized to create a WoO-enabled knowledge base environment. Selecting, binding and running features of virtual world knowledge in the WoO system dynamically generates service knowledge at the service level. Monitoring and analyzing context information, collecting environment status and the user location for VOs and CVOs are the key functionalities in a knowledge creation model. Figure 15 shows the WoO knowledge creation model. The increasing knowledge model provides better observation of the objects for better IoT service creation at the service level.

A virtual world knowledge model represents the relevant behavior of virtual world information, which is predefined according to the domain of the knowledge. Mapping real world information into virtual world information, transforming virtual world information into meaningful virtual world knowledge improves the knowledge-based service quality. Obtaining virtual world knowledge from the virtual world information and adding it to the previous knowledge optimizes the service performance, thus experiencing the virtual world knowledge model that provides appropriate service features. The virtual world knowledge model is represented by rule-based ontologies describing the virtual world objects, such a model provides additional information of the virtual world to the domain experts for defining services. Different attributes of physical devices are defined to create VOs, packaging the VOs with added intelligence in the virtual domain leads to knowledge by reasoning. A service logistic model at the service level is concerned with the part of virtual world service that includes the service knowledge stored in the service information database. Service request at the service level contains relevant VO information for service execution. The functionalities at the service level receive and analyze the service request as well as identify and extract the service parameters, such as VO, CVO information for executing the requested service. The iteration of these processes at a certain period expands the service knowledge in the service logistic model. In the virtual world knowledge creation model, the CVO level interacts with the service level by requesting and replying with service execution where associated VOs take part in the service execution process. Due to the changes in the contextual situation, new service requirements might arrive where the previous experience of the model might not serve as per the nature of the request. Thus virtual world knowledge creation model should have the ability to adapt to the changing environment over time.

### 6.3. Knowledge-Based Service Execution Process

**Figure 16:**
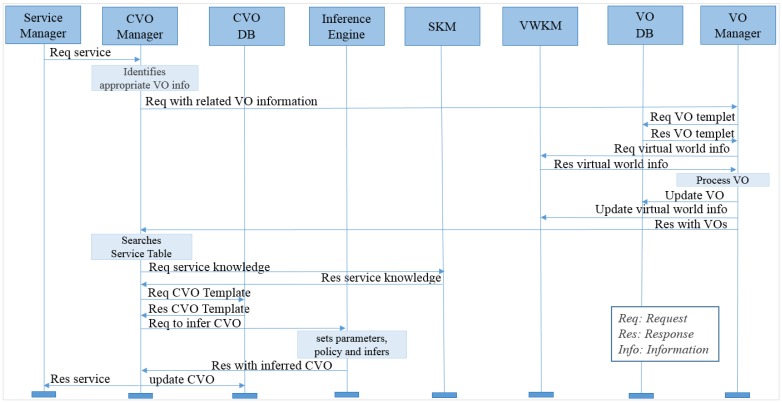
Knowledge-based service execution process.

The knowledge-based services are supported by the WoO knowledge creation model. When a service request arrives from the service manager, the CVO manager identifies appropriate VOs and sends these VOs’ information to the VO manager. The VO manager then collects the VO template from the VO database as well as the virtual world information from the virtual world knowledge model (VWKM). The VO manager processes the associated VOs with virtual world information. The VO manager updates the VO according to the virtual world information as well as updates the virtual world information database. The VO manager then responds to the CVO manager with the VOs. Figure 16 shows the knowledge-based service execution process.

The CVO manager searches for related services based on the service request. For the knowledge-based service execution, the CVO manager requests the service information, which is responded to by the service knowledge model (SKM). Based on the service knowledge model, the CVO manager collects CVO templates from the CVO database. The CVO manager then provides the CVO templates, associated VOs, and service policies to the inference engine for reasoning. The inference engine responds to the CVO manager with the service.

## 7. Prototype Implementation

To realize the knowledge-based IoT services, a use case scenario of an emergency service at a smart shopping mall has been studied. This section discusses the implementation architecture as well as prototype implementation of the use case scenario of the emergency service at a shopping mall. Thus, we have classified different states at the shopping mall, such as normal, cautious, warning and emergency state. A flow diagram has been provided that shows the sequence of the services or actions performed while an emergency state is classified. To create the knowledge-based system, semantic ontology model has been developed.

### 7.1. Classification of States

To provide the emergency service, it is necessary to classify the symptoms and actions for the four distinct states—normal, cautious, warning and emergency state at the shopping mall. Continuous monitoring and analyzing the sensed data classify the predefined states. Comparing and identifying the current state based on the previous history enables the system to decide upon knowledge-based actions.

The application server (AS) monitors sensor values through the gateway and stores them in the database to create knowledge. The ontology server (OS) updates the VOs and CVOs to reflect the changes in the physical devices, such as sensors, CCTV, *etc.* The AS continuously collects the inferred results from the OS for context analysis. The AS classifies the status through the four different CVOs, such as NormalState, CautiousState, WarningState and EmergencyState that are defined for the four distinct states.

The AS analyzes and compares the sensed value with previous data stored in the history database, and if any abnormal reading is found a cautious state will be identified. In case of a continuous increment or decrement of the sensed value over time, the context monitor function identifies the state as a warning state. The knowledge analyzer function searches the history data for matching similar situations to take fast action with the emergency service function to save time. In this case, the AS identifies the nearest guard to the location and sends a notification about the warning state.

**Figure 17:**
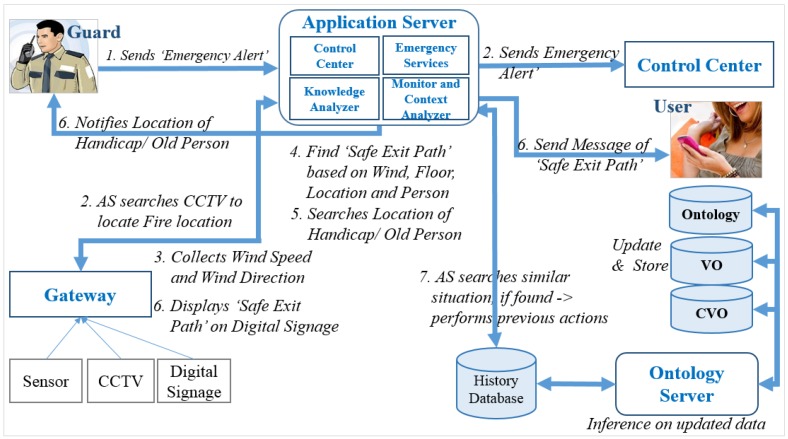
Flow diagram: notification of emergency state and the necessary actions taken by the AS to provide knowledge-based services.

The notification of an emergency state and the actions taken by AS have been shown in Figure 17, where the sequence of actions is represented using numbers. Based on the guard’s confirmation of fire, the emergency service function sends a notification to the control center. The AS displays an emergency message on digital signage. The knowledge analyzer function searches the previous history for similar situations to use a pre-calculated safe exit path for the fast response. The AS analyzes inside and outside weather, wind direction, and fire progress direction to calculate the safe exits. The emergency service function detects handicapped persons and sends a message to the nearest guard regarding those persons’ location as well as provides a safe exit path to the guard and users smartphones. The AS monitors the changes in the context of the fire in order to reconfigure the safe exit path as needed.

### 7.2. Prototype Implementation Architecture

**Figure 18:**
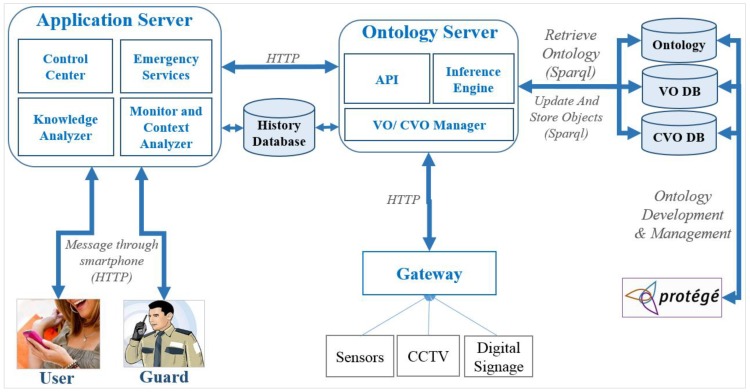
Implementation architecture for the use case scenario.

The implementation architecture is shown in Figure 18. The architecture consists of gateway, AS, OS, history database, VO and CVO databases. The WoO gateway connects to the sensors and actuators with the AS. The AS has four modules including Monitor and Context Analyzer, Emergency Services, Knowledge Analyzer and Control Central. The OS includes API, Inference Engine and VO/CVO Manager. Inference engine applies service rules on the VO and CVO. The OS maintains the VO and CVO databases in order to maintain synchronization with physical devices. The history database is maintained by the AS to keep track of history time to time which might be incorporated in the decision making or providing services in future.

The Monitor and Context Analyzer of the AS reads data periodically from the gateway and sends them to the OS to update VOs and CVOs status. It also sends HTTP GET requests to run reasoning in the OS and gets the results back. It collects additional information, such as users’ locations, guards’ current locations, number of people in the shopping mall, *etc*. Any kind of abnormal situation in the shopping mall is classified by inferencing the related CVOs in OS. In that case, the Emergency Service module is invoked.

The task of the Emergency Services module is pretty straightforward. Based on the shopping mall’s status, it takes some predefined steps. For example, if the shopping mall is in an emergency state this module calculates the safe exits and sends them to the customers, notifies the control center, finds the location of handicapped person, *etc.* The Emergency Services module can request additional services from the Knowledge Analyzer to ensure a faster response such as previously used safe exit paths.

The Knowledge Analyzer module compares the context with the previous history to provide intelligent decision with faster response. For example, if an emergency state is detected there might be a chance to use a previous exit path at the shopping mall. The module can take necessary steps in advance, such as if the shopping mall is in a cautious state that is the early stage of an emergency state, it can calculate safe exit paths in advance based on the early fire symptoms.

In our implementation, the Control Center module basically displays emergency notifications and fire areas using CCTV. It also shows the guards’ positions in the shopping mall. It is possible to select a guard to send notifications or messages or the location of handicapped persons in the shopping mall.

The VO/CVO manager in the OS handles CRUD operations (create, read, update and delete operations) on VOs and CVOs. However, in our implementation we have used only read and update operations for simplicity. The VO/CVO manager provides an interface to the AS using the APIs of the OS. It accepts SPARQL queries from the AS and runs over TDB where VOs and CVOs are stored.

**Figure 19:**
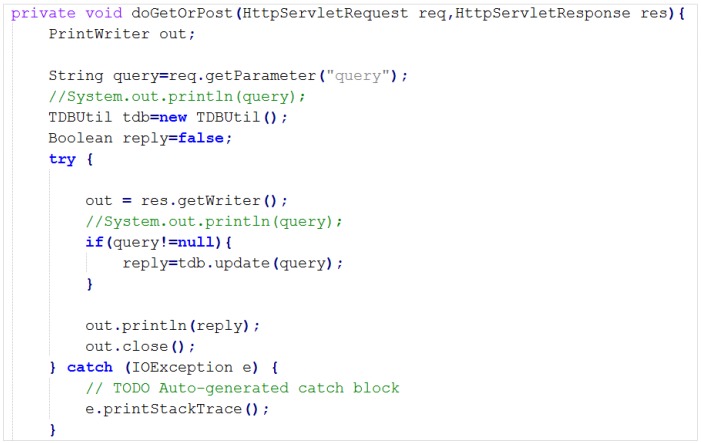
UpdateExec API for updating VO/CVO.

The OS provides different types of APIs to the AS to run different kinds of operations using JAVA servlets, so that AS can send HTTP GET or POST request to OS. Two APIs have been introduced for the VO/CVO manager including UpdateExec (for update query execution) and QueryExec (for reading or getting inferred result). Figure 19 shows a servlet code snippet for updating the API.

### 7.3. Implementation Specifications

At the beginning of the implementation process, a semantic ontology has been designed using Protégé and represented in OWL in the ontology database. The semantic ontology can be achieved by creating linked data and accessing existing databases, thus common format RDF/XML has been used to represent VOs and CVOs in the database. Linked data is the core in the semantic ontology for integrating and reasoning with the large scale of data on the web to exploit knowledge and experience from different datasets. Even though several datasets are available, DBPedia is a large linked dataset that includes content in RDF format as well as incorporates other datasets on the web.

SPARQL has been used to access VOs and CVOs from the OS. To test the server we have used Hermit 1.3.7 as the reasoner in Protégé and Apache Jena as inference engine in the deployment environment. As the semantic ontology is represented in OWL, a Hermit 1.3.7 reasoner determines whether the semantic ontology is consistent or not as well as identifies subsumption relationships among classes, subclasses, data properties, object properties and individuals. The Apache Jena inference engine supports the use of RDF and OWL, which allows deducing facts from current data and defined class descriptions. The Inference Engine also provides an interface through APIs so that AS can request periodically to run the reasoner on updated data. Communication between the AS and gateway as well as AS and OS have been done with HTTP.

### 7.4. Creation of the Semantic Ontology Model

All the sensors are connected to the gateway through ZigBee. Bluetooth has been deployed in the prototype environment. The AS locates customer and guard positions through smartphones via RSSI. AS is developed using Node.js and OS runs on Java. A Java servlet provides an API to the AS. MongoDB has been used to store history for less complexity, where TDB has been used to store the ontology, VOs and CVOs.

The semantic ontology represents the relationship among objects containing different parts that form a composition based on reasoning. The sensors and devices located indoors or outdoors gather data in a specific location. All the sensed data, status of the devices, present condition, customer information and location are recorded in the repository.

**Figure 20:**
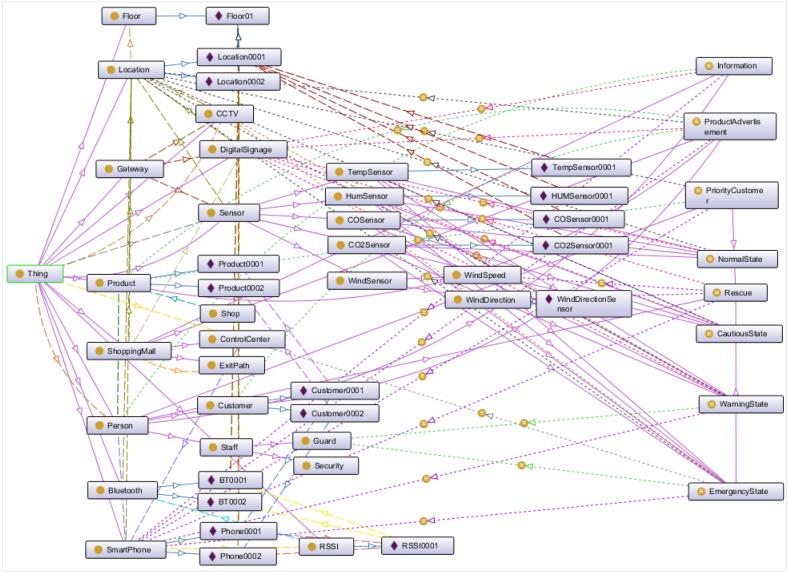
Semantic ontology model for the use case at a smart shopping mall.

Execution of an activity in an emergency situation depends on its location and context. Required services, context and device-oriented activities’ recognition are the fundamental requirements for object virtualization and knowledge base in a semantic ontology. This section discusses the creation process of the semantic ontology model. Figure 20 shows the semantic ontology model for the shopping mall use case that has been designed in Protégé.

At the beginning, domain and scope for development of the semantic ontology were defined. The Sensor (Temperature Sensor, Humidity Sensor, CO Sensor, CO_2_ Sensor, Wind Sensor), Person (Customer, Guard), location, Product, DigitalSignage, SmartPhone and Product are the domain that specify the scope of the ontology. Example of the scope in the ontology includes specifying the status of the shopping mall, such as the normal, cautious, warning or emergency state. Different services, such as emergency service, advertisement of product *etc.* have been provided based on the scope.

All the terms to make statements in the semantic ontology have been listed. In the example semantic ontology, related terms to specify classes are Sensor, Person, Location, Product, ShoppingMall, DigitalSignage, Gateway, CCTV, *etc*. Terms to specify the subclasses have been specified, such as subclasses of Person are*—*Customer, Guard; subclasses of Sensor class are*—*TemperatureSensor, HumiditySensor, WindSensor; subclasses of WindSensor are*—*WindSpeedSensor, WindDirectionSensor. The terms for data properties and object properties also have been specified, such as SensorID, serialnumber, sensorvalue. It is important to get a comprehensive list of terms without worrying about overlap between the concepts they represent, relationships among the terms, or any property that the concepts may have, or whether the concepts are classes or slots.

The semantic ontology model has been developed based on classes and class hierarchy. Class hierarchy has been developed with the definition of the most general concepts in the domain, such as defining with general class—Person. Then specialized Person classes are formed by creating some of its subclasses—Customer and Staff, further categorized Staff class into Guard and Security.

Connecting classes and subclasses form the semantic ontology*,* where data properties and object properties have been used to define the internal structure of the classes that become clots attached to classes. Data properties are used to describe the relationship among instances and data values, such as firstName, lastName, id, age are used to describe the Person class. Object properties are used to describe the relationship among instances of classes. In the semantic ontology, an object property such as hasSmartPhone has been used to relate Person and SmartPhone class, an object property such as hasInterestProduct has been used to relate Person and Product class.

**Figure 21:**
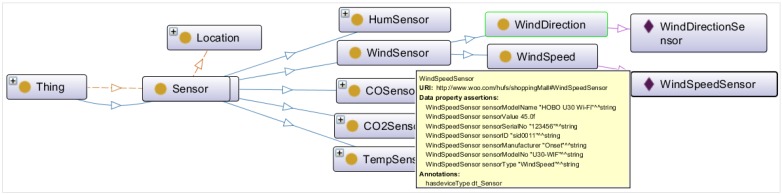
Instances: representation of VOs in the semantic ontology.

Instances represent the VOs in semantic ontology. For example, WindSpeedSensor and WindDirectionSensor are two instances that represent VOs of class WindSensor. Figure 21 shows the representation of VOs of WindSensor class.

**Figure 22:**
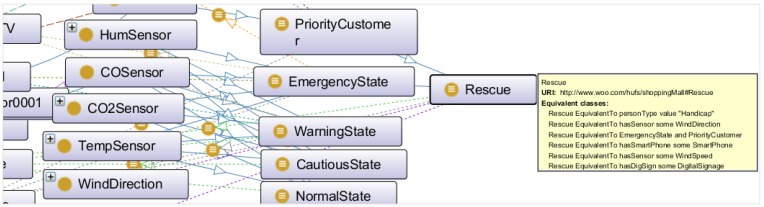
Representation of CVO for service creation.

The goal of the semantic ontology is to integrate, share and merge information for the knowledge-based services, which can be achieved through the creation of CVO. In the use case scenario, CVOs have been defined as classifying states, such as NormalState, CautiousState, WarningState and EmergencyState by reasoning different properties of VOs, CVOs, rules and threshold values that can initiate actions for the normal, cautious, warning and emergency situation, respectively. Other CVOs, such as ProductAdvertisement, PriorityCustomer, Rescue, *etc.* have been defined to offer different services. Figure 22 illustrates the CVO—Rescue, inferred from two CVOs: EmergencyState and PriorityCustomer; five VOs: Person, DigitalSignage, SmartPhone, WindSpeed and WindDirection.

**Figure 23:**
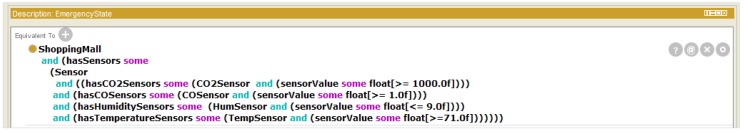
Class expression for EmergencyState CVO defined in Protégé.

Figure 23 shows class expression to create a self-explanatory EmergencyState CVO in the shopping mall. Sensor values are set as predefined thresholds to define a CVO that can be inferred to monitor and analyze the status.

### 7.5. VO and CVO Creation

A defined VO using Protégé can be created using SPARQL at run time. The VO and CVO manager functions of the OS manage the VOs in the ontology using the provided interface to the AS to create, read, update and delete operations in the database.

**Figure 24:**
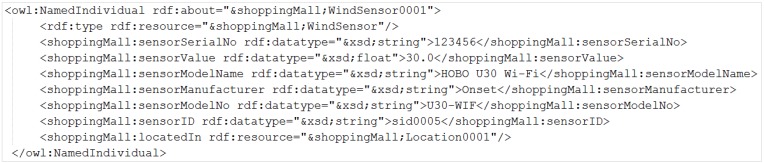
VO description in OWL.

**Figure 25:**
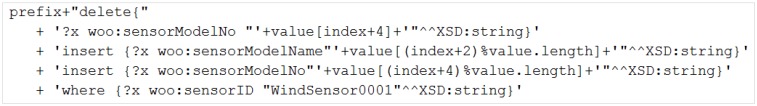
Update of a sensor VO using SPARQL query.

Figure 24 shows an example of the WindSensor VO definition, described as an OWL individual that includes the properties of a specific WindSensor. In the semantic ontology, service-associated VOs are interrelated, thus the AS can extract necessary information using a SPARQL query. VOs are updated using a SPARQL query from the AS to the OS. Figure 25 shows an example SPARQL update query. Sensor values are stored in the history database to be compared by the AS for the knowledge-based service. In order to reduce history database size, valuable data is stored in a time interval. However, more information about a VO can be retrieved from the OS using some properties.

**Figure 26:**
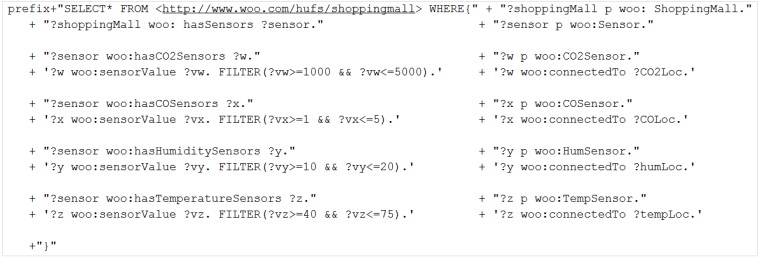
Class expressions into SPARQL query.

Each of the CVOs has its own self-explanatory rules and procedures that identify the CVO. Rules have been defined using class expression in Protégé to create the CVO initially, but later the class expressions are converted into a SPARQL query in the deployment as shown in Figure 26. Even though there are tools for converting class expression in SPARQL, in this implementation we converted manually.

### 7.6. Demonstration on the Use Case Scenario

**Figure 27:**
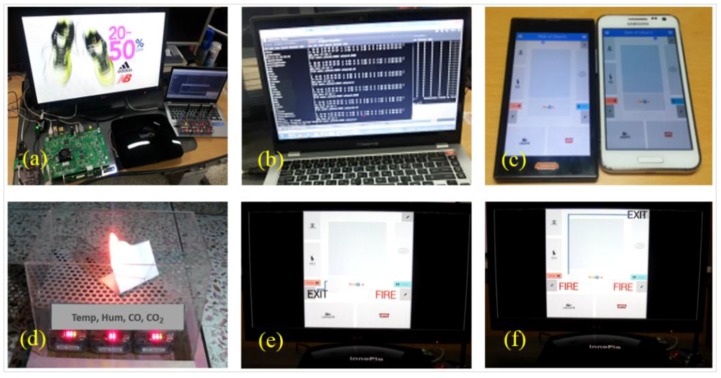
Demonstration of the use case scenario on a WoO service platform, which includes (**a**) smart gateway and sensors; (**b**) Application Server; (**c**) user interface; (**d**) fire occurrence and (**e**,**f**) signage.

The implemented prototype has been demonstrated to realize the WoO-based service platform. Figure 27a–f shows the testbed environment, which includes (a) a smart gateway and sensors; (b) AS; (c) user interface; (d) fire occurrence and (e,f) signage. To provide knowledge-driven emergency services based on the previous history data, we have defined related VOs, such as Sensors, Gateway, and DigitalSignage, as well as CVOs, including NormalState, CautiousState, WarningState and EmergencyState using the semantic ontology in the prototype.

In the demonstration, different sensors, such as temperature, humidity, CO and CO_2_ were used to sense the current status. All the sensors and signage were connected to the gateway (Figure 27a). The sensors periodically sensed and sent context information to the AS (Figure 27b) through the gateway. In the case of an emergency situation, the AS provided safe exit paths to guards’ and users’ smartphones (Figure 27c) as well as displayed on the computer screen as digital signage.

For testing the environment, we created a fire where four types of sensors were set earlier (Figure 27d). Sensors sensed current data and sent the data to the gateway. Due to the increase in temperature, CO, CO_2_ and decrease in humidity value for a certain period, the AS identified the state of warning as triggered by the OS according to the predefined threshold values and functionalities. The AS notified the guards’ smartphones for checking and confirming the fire. We confirmed the fire using the guard application interface. Based on the fire confirmation, the emergency state was identified, then the AS provided safe exit paths that were sent to the guards’ and users’ smartphones as well as displayed on the signage (Figure 27e). To test the environment further, another fire was created at a different location on the same floor. Just as it occurred earlier, a notification was sent to a guard’s smartphone to check and confirm. Based on the confirmation of the fire, the AS reconfigured and provided safe exit paths to guards’ and users’ smartphones and displayed them on the signage (Figure 27f).

## 8. Conclusions

Virtualizing real world physical devices enables virtual representation of features and capabilities of objects in the virtual world to perform operations on the WoO platform. Collecting and converting the raw data into meaningful information and connecting the relation of information in order to form knowledge provides intelligence to the IoT environment.

Knowledge-based composition and orchestration provide application-level intelligent services on a WoO platform. How the knowledge creation model synchronizes the virtual world knowledge model and service logistic model that make possible knowledge-based services has been discussed. Knowledge-based service execution processes based on a knowledge creation model have also been presented.

A semantic ontology-based WoO platform monitors the real time context and analyzes users’ preferences based on previous history data to respond with intelligent decisions and services. A semantic ontology model in terms of VOs and CVOs to create a context-aware knowledge-based service and an implementation architecture on a use case of an emergency service have been designed and discussed. The implementation processes for creating the semantic ontology using OWL, as well as defining VOs in RDF and creating updates using SPARQL have been presented. Finally, the implemented prototype has demonstrated that it can successfully classify the emergency state and provide services in a use case scenario.

Even though the prototype that has been implemented could demonstrate that it performed well, the performance of the system has not been evaluated in this paper. Currently, we have been working on the enhancement of the composition of VO, so that all the functional entities can be implemented on the WoO platform; we are thus motivated to evaluate the performance on the system requirements, as well as restrictions in the processing capacity and scalability issues with service composition in our future work.
